# A Detour Task in Four Species of Fishes

**DOI:** 10.3389/fpsyg.2018.02341

**Published:** 2018-11-29

**Authors:** Valeria Anna Sovrano, Greta Baratti, Davide Potrich

**Affiliations:** ^1^Center for Mind/Brain Sciences, University of Trento, Rovereto, Italy; ^2^Department of Psychology and Cognitive Science, University of Trento, Rovereto, Italy

**Keywords:** fish, detour task, goal-object, object permanence, comparative psychology

## Abstract

Four species of fish (*Danio rerio*, *Xenotoca eiseni*, *Carassius auratus*, and *Pterophyllum scalare*) were tested in a detour task requiring them to temporarily abandon the view of the goal-object (a group of conspecifics) to circumvent an obstacle. Fishes were placed in the middle of a corridor, at the end of which there was an opaque wall with a small window through which the goal was visible. Midline along the corridor two symmetrical apertures allowed animals to access two compartments for each aperture. After passing the aperture, fishes showed searching behavior in the two correct compartments close to the goal, appearing able to localize it, although they had to temporarily move away from the object’s view. Here we provide the first evidence that fishes can solve such a detour task and therefore seem able to represent the “permanence in existence” of objects, which continue to exist even if they are not momentarily visible.

## Introduction

Köhler (1925b) defined “problem” as the unreachability of a goal-object by using direct routes due to the presence of an obstacle. It is thus necessary to circumvent the obstacle, by temporarily moving away from the target. This type of solution of a peculiar environmental challenge introduced what is known nowadays as the “detour problem.” For example, in the problem of detour reported by Barash (1977), dogs and squirrels had to circumvent a pole, in order to reach a bowl of food. Typically, dogs pulled the leash, whimpered, run in disorder, some of them even fell asleep and after waking up they started again from the beginning. Contrarily, squirrels solved the problem immediately.

Probably, there are several factors that could hinder the solution of the detour problem. One of these factors could be the peculiarity of the evolutionary adaptation niches: for example, squirrels moving from a branch to another one usually carry out detour. Moreover, perceptual factors could act as an impediment: it is essential that an obstacle is perceived as one (for example, in some species of birds vertical objects – like blades of grass, light shrubs, etc. – often do not constitute obstacles). Finally, motivational factors could play a role in the detour task: the more visible the object is behind a barrier, the more time the animals can take to solve the problem, because it is more difficult to inhibit the tendency to reach the goal directly instead of adopting a detour behavior. This is particularly accentuated in detour tasks in which the goal is constantly visible, where animals immediately try to join the salient visible object (Köhler, 1925a,b; for a review see Vallortigara et al., 2002; Kabadayi et al., 2018).

Another very important aspect in detour problems is related to the sensory availability of the target to sensory information. During the phase in which it is necessary to get around the obstacle, the animal may temporarily lose every sensory contact with the goal. If the animal is unable to keep in mind the sensorially disappeared object, it would not be able to solve the problem. The concept of “object permanence” has been studied by Piaget in human babies. Infants up to 8 months, after observing an object that has been covered did not seek for it, as if it did not exist anymore (Piaget, 1936). However, more recent research showed that 5-month-old babies already know that when an object is hidden it does not cease to exist (Baillargeon et al., 1985). This concept of “object permanence” has been widely investigated also in animal species (e.g., the most recent reference in great apes: Neiworth et al., 2003; in dogs: Gagnon and Doré, 1994; in cats: Goulet et al., 1994; in golden hamsters: Thinus-Blanc and Scardigli, 1981; in birds: Vallortigara et al., 1998; Regolin et al., 2005; Zucca et al., 2007).

Detour abilities have been studied mainly using two paradigms: “continuously visible goal” and “initially visible goal” tasks (Kabadayi et al., 2018). In the continuously visible paradigm, the goal is visible through the obstacle (a transparent object) during the animals’ detour response. Conversely, in the initially visible paradigm, the goal is visible from the animals’ starting position but becomes not accessible during detour, because of an occlusion (an opaque barrier).

In literature there are several studies with vertebrate species that were dealing with detour, both using continuously and initially visible goal paradigm (for a review, see Kabadayi et al., 2018). Nevertheless, these two paradigms differ also for the setup: continuous tasks, for example, use a transparent object, such as a cylinder, a V-shaped barrier or a square box; on the other hand, initially visible goal tasks use an opaque barrier that prevents the view of the goal while animals are detouring, for example the four-compartments box. In particular, this task requires subjects to turn their back to the goal-object and choose among four compartments, where only two lead to it (Kabadayi et al., 2018).

In the initially visible goal tasks different cognitive mechanisms are involved, such as inhibitory control, working memory, route planning, and object permanence. Inhibitory control allows animals to inhibit the tendency to reach directly the visible goal-object during detour (Moll and Kuypers, 1977; Diamond, 1990). Intuitively, the more visible the goal behind the obstacle is, the more difficult is the detour response. On the other hand, working memory, route planning, and object permanence are probably essential when the goal-object becomes invisible: animals have to retrieve from the working memory a representation of the goal (or of the goal’s position) and plan the most effective detour routes to join it (see Regolin et al., 1994).

Detour abilities have been studied in several species of non-human mammals using different setups (primates: Köhler, 1925a,b; dogs: Wyrwicka, 1959; Chapuis et al., 1983; Fiset et al., 2007; cats: Poucet et al., 1983; horses: Baragli et al., 2011; hamsters: Vauclair, 1980; Chapuis and Scardigli, 1993; rats: Blancheteau and Le Lorec, 1972; marsupials: Wynne and Leguet, 2004). Moreover, the ability to solve detour problems has been well demonstrated in birds (Regolin et al., 1995a,b; Zucca et al., 2005). Similarly, fish tested in an apparatus with L- or T-shaped barriers (transparent or opaque) showed detour abilities as other vertebrates assessed through the same paradigm and setup (continuously visible goal: Beniuc, 1938; Schiller, 1948; Bisazza et al., 1997; Facchin et al., 1999; Reddon et al., 2009; Moscicki et al., 2011; Lucon-Xiccato et al., 2017; initially visible goal: Russell, 1931; Zunini, 1938; Schiller, 1949).

In a four-compartments box task conducted by Regolin et al. (1995b), 2-days old chicks were able to reach the imprinting object located behind a barrier, by entering one of the correct corridors (and so keeping in mind a sort of representation of the goal), although they temporarily moved away from the object’s view. Two-days old chicks solved the task and therefore seemed to be able to represent the “permanence in existence” of the imprinting object: in other words, their goal-object continued to exist even if it was not momentarily visible.

However, the ability to maintain an internal representation of the goal could not be the only possible explanation to successfully detours. An alternative and more parsimonious idea comes from a study carried out by Walker and Miglino (1999) with robots, which duplicated the results by Regolin et al. (1995b) using the same setup. They evolved artificial organisms making simple detours based on the inputs detected by the proximity sensors. Robots, in absence of any kind of internal representation, showed analogous performance obtained in chicks. These authors suggested that detouring could emerge from “primitive” forms of exploratory behavior and taxis (for example, moving toward a target following an obstacle until it goes out of sight). Nevertheless, this explanation does not exclude that chicks could use more sophisticated cognitive strategies to solve a detour task.

Using a similar detour paradigm, Zucca et al. (2005) tested three different bird species (quails *Coturnix coturnix*, herring gulls *Larus cachinnans*, and canaries *Serinus canaria*) in the same four-compartments box, finding that the adaptation to different ecological niches is the most likely factor that explains the differences in detour performance.

**FIGURE 1:**
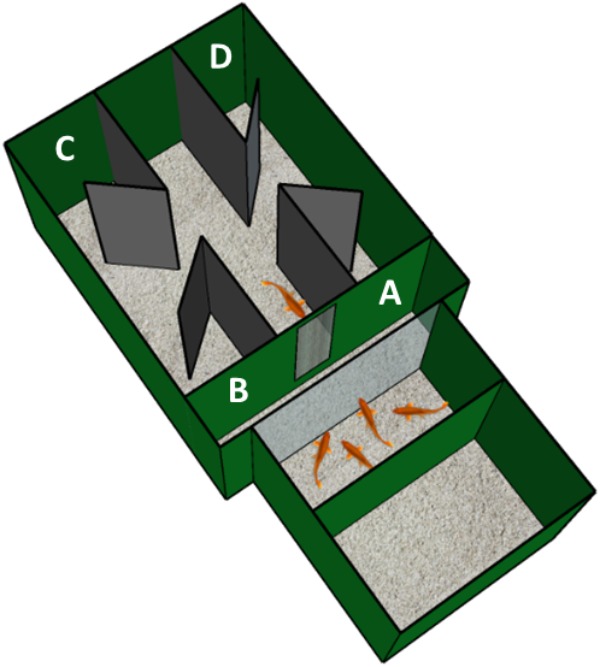
Experimental apparatus. Schematic representation of the experimental apparatus used for the detour task with fish. It is composed of two adjacent glass tanks, covered with dark green plastic material (Poliplak^®^): the smaller tank (on the bottom right) housed four adult fish that acted as social attractor for the experimental subject, instead located in the larger tank (on the upper left). Furthermore, the drawing shows the grid through which the experimental subject could observe the goal-object (social stimuli) and the four compartments, placed at the four open ends of the corridor: **A** and **B**, the correct compartments, localized close to the grid; **C** and **D**, the incorrect compartments, localized on the opposite side of the experimental tank.

The aim of our experiment is to extend the results in a detour task never used in fish, the four-compartments box, providing a direct comparison with other vertebrate species (Regolin et al., 1995b; Zucca et al., 2005). Indeed, we set up an experimental condition in a rectangular apparatus, a four-compartments box, consisting of two adjacent tanks: one of the two tanks housed a group of conspecifics, as a goal-objects, while the other tank was divided into a corridor and four compartments of choice, where the two correct compartments (see Figure 1) were those close to the goal. Fishes were placed in the middle of the corridor, at the end of which there was an opaque wall with a small window through which the goal was visible. Midline along the corridor, two symmetrical apertures allowed fishes to access to the four compartments.

The choice for the two closest compartments to the goal could have been an indicator of the capacity to solve the detour task, although the animals had to temporarily move away from the object’s view; on the contrary, the choice for the furthest compartments could have highlighted the incapacity to solve the detour task, with a consequent absence of the ability to keep in mind the goal to be searched.

In attempt to evaluate potential interspecific peculiarities based on the ecology of animals, four different freshwater species of fish were considered: *Xenotoca eiseni* (Goodeidae), *Danio rerio*, *Carassius auratus* (both Cyprinidae), and *Pterophyllum scalare* (Cichlidae). They belong to different families (Goodeidae, Cyprinidae, Cichlidae) and are native to distant geographical areas (respectively, Central America, South Asia, East Asia, South America), adapting to various ecosystems (rivers, canals, lakes, swamps). These four species are also animal models employed in manifold cognitive tasks (spatial, perceptual, numerical) (the most significant and recent references: Vargas et al., 2004; Lee et al., 2012; Stancher et al., 2013; Potrich et al., 2015; Gómez-Laplaza and Gerlai, 2016; Sovrano et al., 2016).

Although previous studies found species-specific differences on the four-compartments box task (Zucca et al., 2005), we did not expect any difference in performance among the four species used in our experiment. Differently than bird species, adapted to inhabit aerial or terrestrial environments, fish live in similar aquatic niches, thus facing the same potential obstacles (e.g., algae, rocks). Moreover, by properly solving a task of circumventing obstacles may be fundamental for survival, coming to constitute a common cognitive tool among organisms, regardless of their phylogenetical position and the peculiar environments where they have adapted.

## Materials and Methods

### Subjects

The subjects were 149 adult fishes belonging to four different freshwater species (*X. eiseni*, *N* = 39; *D. rerio*, *N* = 41; *C. auratus*, *N* = 39; *P. scalare*, *N* = 30). As social stimulus and goal-object, four adult fish belonging to the same species of the experimental subjects were used. Fish were maintained in their home tanks (25 l), enriched with gravel to ensure a comfortable habitat and cleaned with suitable filters (Aquarium Systems Duetto 100, Newa, I). The water temperature was maintained at 26°C. Fish were fed twice a day with dry food (GVG-Mix, Sera^®^ GmbH, D).

### Ethics Statement

The present research was carried out at the Animal Cognition and Neuroscience Laboratory (ACN Lab) of the CIMeC (Center for Mind/Brain Sciences), at the University of Trento (Italy). All husbandry and experimental procedures complied with European Legislation for the Protection of Animals used for Scientific Purposes (Directive 2010/63/EU) and were previously authorized by the University of Trento’s Ethics Committee for the Experiments on Living Organisms, and by the Italian Ministry of Health (auth. num. 1111/15-PR, prot. num. 13/2015).

### Apparatus and Materials

The apparatus (Figure 1) was an adaptation of the apparatus used with chicks in a similar detour task (Regolin et al., 1995b) and consisted of two glass tanks facing each other: the larger one (35 cm × 30 cm × 28 cm) housed the experimental subjects, while the smaller one (25 cm × 25 cm × 25 cm) housed the social stimuli, used as goal-objects. Both tanks were internally covered on three sides with dark green plastic (Poliplak^®^), leaving uncovered the two adjacent walls (and so, permitting to the experimental subject to see the social stimuli). The floor of both tanks was homogeneously covered with gravel (3 cm in depth) and the water level was equal in both tanks (19 cm in high): these measures made sure that fish experienced a visual continuity of the two tanks. The water temperature was maintained constant at 26°C, as in home-tanks, with the aid of a heater, and two filters (316, Eden^®^, present in not-experimental phase) ensured good water quality.

In the tank housing the social stimuli a compartment made of dark green plastic (Poliplak^®^, 25 cm × 25 cm × 4 cm) was placed. In this compartment four adult fish were confined in order to attract the experimental subject to reach the goal. The social stimuli were located 15 cm away from the barrier. Inside the experimental tank (see Figure 1) there was a corridor made of two black plastic walls (Poliplak^®^, 24 cm in length, 22 cm in height, spaced 9 cm apart). At the end of the corridor facing the social goal-stimuli, there was a dark green panel with a rectangular aperture (5 cm × 14 cm) with a thick grid (0.2 mm). Through the aperture, the social stimuli were visible from the experimental fish in an “observation area” (9 cm × 25 cm), in which the subject was confined by placing a removable sliding dark green panel (placed 9 cm away the aperture). After 3 min of observation the sliding door was opened allowing the subject to go outside the corridor, into the experimental area: in the midline along the corridor there were two symmetrical apertures (4 cm in size). Four diagonal partitions made of black plastic (Poliplak^®^, 5 cm × 22 cm, with an acute angle of 45°) were localized outside the corridor, offering the fish the possibility to choose among four compartments, two for each aperture (correct compartments: A and B; incorrect compartments, C and D, in Figure 1). The partitions prevented the animals from being faced immediately with the closed walls after exiting the corridor, in order to avoid a possible condition that would have inhibited the searching behavior, thus guaranteeing a genuine choice.

The experimental apparatus was placed in a darkened and acoustically isolated room and lit centrally from above (30 cm from the apparatus) by a white LED light bulb (3 W) to create a soft illumination of the environment and a central webcam (LifeCam Studio, Microsoft), fixed on the top, recorded the fish behavior in the area of choice (four compartments: A, B, C, D).

### Procedure

Each fish performed four trials in two consecutive days, consisting of one single choice for each trial. In each trial, the subject was confined in the “observation area” of the corridor using a fish net, paying particular attention to release the fish facing the grid, where the social stimuli in the adjacent tank were visible through it. Each fish remained into the observation area for a period of 3 min. After the observation time, the sliding panel was gently raised and the subject was left free to make its choice for a maximum time of 10 min. A choice was considered done when the entire body of the fish entered one of the four compartments. At the end of the trial, the subject was again confined in the “observation area” for the next trial, in order to collect two valid choices per day.

The first absolute choice and the number of choices for the four compartments, i.e., total four choices per fish summed over the two trials of two sessions, were used as individual data. An inter-observer reliability criterion (Caro et al., 1979) was applied in the re-coding of a subset of 10% of different videos (*p* < 0.001, Pearson’s correlation between the ratio calculated on the original coding and on the *de novo* coding performed by an experimenter blind on the test condition of the fish).

### Data Handling

The mean number of choices and the first absolute choice for each compartment has been considered, in order to evaluate whether there was a difference for sectors A-B (cumulating the choices made in sector A plus B: the correct compartments) versus sectors C-D (cumulating the choices made in sector C plus D: the incorrect compartments).

When the total of the four choices collected in 2 days was considered, data were analyzed by the analysis of variance (ANOVA) with species as a between-subjects factor, and compartments (A-B vs. C-D) and time (1st vs. 2nd day of test) as within-subjects factors. To estimate the effect sizes, partial eta-squared ($\eta_{p}^{2}$ ) as the index for ANOVA was reported. In order to compare A vs. B and C vs. D a paired Student’s *t*-test was applied.

On the other hand, when considering only the first choice at the detour test, data were analyzed by Chi-squared test, considering both each species individually and all the species together.

Data were analyzed with the IBM SPSS Statistics 24.0 software package.

## Results

### Analysis of Detour Trials

**FIGURE 2:**
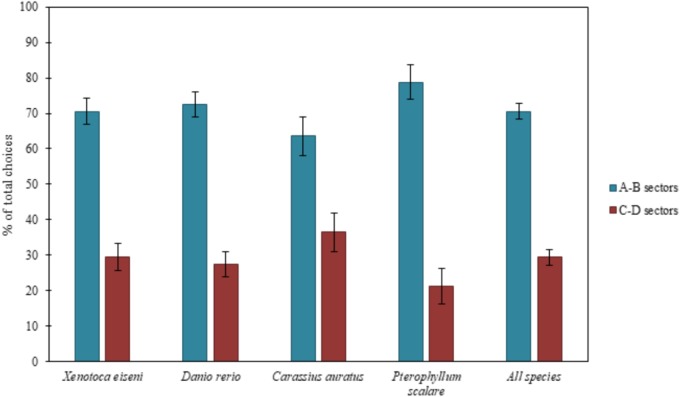
Results of the total choices. The bars graph shows the percentages of choices (group means ± SEM are shown) for the two correct compartments (A-B) versus the two incorrect compartments (C-D) of the experimental apparatus, considering all species both separately (*X. eiseni*, *D. rerio*, *C. auratus*, *P. scalare*) and together. Four species of fish indistinctly were able to solve the detour task, choosing predominantly both the two correct compartments (A-B) close to the goal rather than the incorrect ones (C-D) far from the goal: moving away momentarily from the goal, they seem to be able to keep in mind the object to reach.

Results are reported in Figure 2, considering all species both separately and together.

Four of the 39 *C. auratus* and ten of the 30 *P. scalare* failed to exit the corridor within 10 min for all of the four test trials, showing attraction for the social stimuli visible behind the grid and the consequent tendency to still remain in the “observation area.” Moreover, the peculiar locomotor activity of angelfish (*P. scalare*) could be another factor: typically they swim at low speed, in order to appear less conspicuous to predators (Blake, 1979). In these sporadic cases, we considered only their first absolute choice and we proceeded with the statistical analysis of total choices on the remaining animals.

The ANOVA, applied on the total of the four choices (*N* = 135), with species (*X. eiseni*, *D. rerio*, *C. auratus*, *P. scalare*), as a between-subjects factor, and compartments (A-B vs. C-D) and time (1st vs. 2nd day of test), as within-subjects factors, revealed a significant effect of compartments [*F*(1,131) = 85.77 *p* ≤ 0.0001, $\eta_{p}^{2}$ = 0.396]. There were no other statistically significant effects [time: *F*(1,131) = 0.36; species: *F*(3,131) = 0.95; time × species: *F*(3,131) = 0.95; compartments × species: *F*(3,131) = 1.68; time × compartments: *F*(1,131) = 0.02; time x compartments × species: *F*(3,131) = 0.64].

The paired Student’s *t*-test applied in order to compare A vs. B and C vs. D did not reveal statistically significant differences between the two correct compartments as well as the two incorrect compartments, demonstrating a balance of choices between the two pairs of sectors [total choices: A vs. B: *t*(134) = 0.32 *p* = 0.75; C vs. D: *t*(134) = 0.8 *p* = 0.43].

The Chi-squared test applied on the first choices (*N* = 149) showed the following results: considering the four species separately, 28 *X. eiseni* chose the correct compartments A-B (χ^2^= 7.410 *df* = 1 *p* = 0.006), 24 *D. rerio* (χ^2^= 1.195 *df* = 1 *p* = 0.274), 26 *C. auratus* (χ^2^= 4.333 *df* = 1 *p* = 0.037), and 26 *P. scalare* (χ^2^= 16.133 *df* = 1 *p* ≤ 0.0001). Considering all the species together, 104 fishes chose the correct compartments A-B (χ^2^= 23.362, *df* = 1, *p* ≤ 0.0001). There were no significant differences between the two correct compartments A and B (*X. eiseni*: 15 vs. 13, *p* = 0.705; *D. rerio*: 14 vs. 10, *p* = 0.414; *C. auratus*: 14 vs. 12, *p* = 0.695; *P. scalare*: 14 vs. 12, *p* = 0.695; all species: 57 vs. 47, *p* = 0.327) and between the two incorrect compartments C and D (*X. eiseni*: 7 vs. 4, *p* = 0.366; *D. rerio*: 8 vs. 9, *p* = 0.808; *C. auratus*: 9 vs. 4, *p* = 0.166; *P. scalare*: 2 vs. 2, *p* = 1; all species: 26 vs. 19, *p* = 0.297), demonstrating also in the absolute first choice a balance between the two pairs of sectors in all species.

When considering the total amount of choices for the compartments, the four species of fish proved to be able to choose predominantly the two correct compartments (A-B) with respect to the incorrect ones (C-D). When considering the absolute first choices of fishes, only zebrafish (*D. rerio*) did not seem able to correctly choose the correct compartments from the first attempt, probably due to their peculiar behavioral pattern: they usually show high levels of motility and boldness, especially in an environment never explored before (Kalueff et al., 2013). The other three species fully confirmed the results of the total choices, which seemed to be, for zebrafish too, a more accurate variable in the description of fish behavior at this test.

## Discussion

Our work aimed to assess the ability of four fish species in the solution of an initially visible detour task using a four-compartments box setup, similar to the one used with avian species (Regolin et al., 1995b; Zucca et al., 2005) and robots (Walker and Miglino, 1999). Here we provide the first evidence that also fishes, as well as birds, solve a four-compartments box detour task, choosing the two compartments closer to the goal (A-B).

A parsimonious idea suggests that the detour ability could emerge from “primitive” forms of exploratory behavior and taxis (Walker and Miglino, 1999). Alternative explanations refer instead to a trial and error strategy (Redish, 2016) or a stimulus-response chain (Grush, 1997; Cross and Jackson, 2016). All these mechanisms probably do not need an internal representation. On the other hand, more sophisticated strategies can not be excluded in the solution of similar tasks. Animals could have also kept the vanished object in mind to solve the problem. In fact, in our experiment, in order to reach a goal, animals must temporarily move away from the goal-object’s view, showing that the goal has not gone “out of existence” when it can no longer be seen. Fish would seem able to represent the “permanence in existence” of objects (at least of the object to be reached and lost sight for a few moments), which continue to exist although they are not momentarily visible.

The fact that we have not found great differences among the four fish species is not unexpected and could be understandable in terms of adaptation: the ability to circumvent a potential obstacle, in order to reach an important target, changing direction and temporarily moving away from the goal, could probably be a widespread necessity among animals to fulfill the normal demands of life on our planet. The ability to circumvent obstacles could, in fact, be crucial when an object relevant for survival has to be achieved, such as when a predator is hunting, but also when an animal is escaping from a potential predator. Making detours could be crucial both for prey and predators: a predator will have excellent ability of detour (Cross and Jackson, 2016). This does not exclude that also prey are able to circumvent obstacles to easily escape from the predator. When a predator develops peculiar skills, the prey develops concurrent cognitive strategies to contrast them.

Nevertheless, the fact that differences in detour performance in some vertebrate species have been reported (for example, by Barash, 1977; Zucca et al., 2005), raises issues related to several factors that can hinder the solution to detour tasks: the peculiarity of the evolutionary adaptation niches, perceptual or motivational factors could play a determining role in success or failure of the task’s solution (Köhler, 1925a,b; for a review see Vallortigara et al., 2002). This does not find its further reply here, suggesting that several species of fish underwent the same ecological pressures and needs.

Nevertheless, some fish were visually attracted to the reward behind the grid (mainly *P. scalare*) without turning their back to the reward and execute the detour task. Typically, *P. scalare* species has a reduced locomotor activity, in order to appear less conspicuous to predators (Blake, 1979). This could be an explanatory factor for its preference to remain in the “observation area.” On the other hand, a cognitive mechanism could be recruited, such as inhibitory control, which allows animals to inhibit the tendency for a direct reach of the visible goal-object during detour behavior (Moll and Kuypers, 1977; Diamond, 1990). However, it is not clear to what extent inhibitory control is involved in the different species.

Despite our evidence, by extending the investigation to other species of fish adapted to different environments from those reported here, such as seawater habitats, it could perhaps shed light at least on the role of peculiarity of the evolutionary adaptation niches. In fact, it has been shown that marine fish can show different behavioral patterns compared to those of freshwater (Sovrano et al., 1999, 2001; Sovrano and Andrew, 2006; Besson et al., 2017).

In conclusion, our findings add evidences about the ability to solve a detour task and to represent an alleged “permanence in existence” of objects even in freshwater fish. From a comparative point of view, it seems likely that this cognitive skill is maintained in species inhabiting different environments (both terrestrial and aquatic): perhaps under different selective pressures, populations belonging to peculiar ecological niches have offered common “intelligent” solutions to similar problems.

## Author Contributions

VS conceived the study. VS and DP designed it together. DP and GB performed the experiments and contributed to materials and animal care. VS and GB analyzed and interpreted the results. VS, DP, and GB wrote the paper.

## Conflict of Interest Statement

The authors declare that the research was conducted in the absence of any commercial or financial relationships that could be construed as a potential conflict of interest.
